# Improving portable low-field MRI image quality through image-to-image translation using paired low- and high-field images

**DOI:** 10.1038/s41598-023-48438-1

**Published:** 2023-12-01

**Authors:** Kh Tohidul Islam, Shenjun Zhong, Parisa Zakavi, Zhifeng Chen, Helen Kavnoudias, Shawna Farquharson, Gail Durbridge, Markus Barth, Katie L. McMahon, Paul M. Parizel, Andrew Dwyer, Gary F. Egan, Meng Law, Zhaolin Chen

**Affiliations:** 1 Monash Biomedical Imaging, Monash University, Melbourne, VIC Australia; 2 Department of Data Science and AI, Faculty of Information Technology, Monash University, Melbourne, VIC Australia; 3 Department of Neuroscience, Central Clinical School, Monash University, Melbourne, VIC Australia; 4 Department of Radiology, Alfred Hospital, Melbourne, VIC Australia; 5 Australian National Imaging Facility, Brisbane, QLD Australia; 6 Herston Imaging Research Facility, University of Queensland, Brisbane, QLD Australia; 7 School of Information Technology and Electrical Engineering and Centre for Advanced Imaging, University of Queensland, Brisbane, QLD Australia; 8 School of Clinical Science, Herston Imaging Research Facility, Queensland University of Technology, Brisbane, QLD Australia; 9 David Hartley Chair of Radiology, Department of Radiology, Royal Perth Hospital, Perth, WA Australia; 10 Medical School, University of Western Australia, Perth, WA Australia; 11 South Australian Health and Medical Research Institute, Adelaide, SA Australia

**Keywords:** Biomedical engineering, Brain imaging

## Abstract

Low-field portable magnetic resonance imaging (MRI) scanners are more accessible, cost-effective, sustainable with lower carbon emissions than superconducting high-field MRI scanners. However, the images produced have relatively poor image quality, lower signal-to-noise ratio, and limited spatial resolution. This study develops and investigates an image-to-image translation deep learning model, LoHiResGAN, to enhance the quality of low-field (64mT) MRI scans and generate synthetic high-field (3T) MRI scans. We employed a paired dataset comprising T1- and T2-weighted MRI sequences from the 64mT and 3T and compared the performance of the LoHiResGAN model with other state-of-the-art models, including GANs, CycleGAN, U-Net, and cGAN. Our proposed method demonstrates superior performance in terms of image quality metrics, such as normalized root-mean-squared error, structural similarity index measure, peak signal-to-noise ratio, and perception-based image quality evaluator. Additionally, we evaluated the accuracy of brain morphometry measurements for 33 brain regions across the original 3T, 64mT, and synthetic 3T images. The results indicate that the synthetic 3T images created using our proposed LoHiResGAN model significantly improve the image quality of low-field MRI data compared to other methods (GANs, CycleGAN, U-Net, cGAN) and provide more consistent brain morphometry measurements across various brain regions in reference to 3T. Synthetic images generated by our method demonstrated high quality both quantitatively and qualitatively. However, additional research, involving diverse datasets and clinical validation, is necessary to fully understand its applicability for clinical diagnostics, especially in settings where high-field MRI scanners are less accessible.

## Introduction

Magnetic resonance imaging (MRI) is a non-invasive medical imaging modality that can comprehensively visualize tissues and organs, exhibits superior soft-tissue contrast relative to alternative imaging modalities and can demonstrate subtle pathologies^1^. MRI utilises a strong magnetic field, radiofrequency pulses, and sophisticated computational algorithms to collectively generate diagnostic images of many body regions, including the brain, spine, organs, and joints. A notable feature of MRI is its absence of ionizing radiation, which is associated with reduced radiation-related risks^2^. Low-field MRI provides opportunities to develop a more compact, cost-effective, and portable system in comparison to current clinical MRI scanners ($\ge$1.5T field strengths)^3^. Although low-field MRI images suffer from reduced signal-to-noise ratio (SNR) compared to their high-field counterparts, low-field MRI is of potential interest for a number of imaging applications, including musculoskeletal and neuroimaging. Point of care (POC) MRI for use in the emergency department (ED) or intensive care unit (ICU) settings could serve as a viable alternative in remote or economically challenged areas in the world^4–8^.

The recent advent of portable low-field MRI scanners, such as the 64mT Hyperfine Swoop, holds significant promise for reducing MRI access inequality with acceptable diagnostic quality^9–15^. However, the limited spatial resolution and SNR of low-field MRI precludes using traditional image analysis tools, such as the FMRIB automated segmentation tool, FSL-FAST^16,17^. As a result, there has been a growing interest to determine whether novel methods can translate images acquired with low-field POC MRI scanners to be comparable to those obtained with high-field scanners^18,19^. The method, known as image-to-image translation, holds potential to improve the diagnostic value of images acquired using low-field scanners. Recently, deep learning-based (artificial intelligence) approaches, have shown significant promise for medical image synthesis^20–24^. Several state-of-the-art methods previously used for natural image-to-image translation have been adapted to perform low-field to high-field MRI image-to-image translation^9,17,25,26^. These include generative adversarial networks (GANs), CycleGAN, U-Net, multi-scale fusion networks, conditional generative adversarial networks (cGAN), and Pix2Pix^27–31^.

Based on U-Net^29^, Iglesias et al.^9^ proposed a state-of-the-art synthetic super-resolution method called SynthSR. The method employs a network to generate 1 mm isotropic T1-weighted structural images from clinical MRI scans with varying orientation, resolution (including low-field), and contrast. This innovative approach can potentially advance quantitative neuroimaging in both clinical care and research settings^32^. Subsequently, Iglesias et al.^33^ provided a proof-of-concept (SynthSeg) for applying the SynthSR method to perform quantitative brain morphometry analysis on low-field MRI data. The results demonstrate that portable low-field MRI can be enhanced with SynthSR to yield brain morphometric measurements that correlate with those obtained from high-resolution images. More recently, a robust version of the Iglesias et al.^33^ method called SynthSeg$^+$, with robustness for any MRI resolution and contrast, was proposed by Billot et al.^34^ who subsequently investigated its performance on clinical scans^35^. Similarly, Laguna et al.^17^ introduced an image-to-image translation architecture inspired by CycleGAN^28^, which integrates denoising, super-resolution, and domain adaptation networks to address the challenges of portable low-field MRI in terms of resolution and signal-to-noise ratio.

The aforementioned adaptive methodologies motivate the current investigation into the performance of advanced image-to-image translation models for low-field MRI applications with a particular focus on their ability to maintain diagnostic integrity and preserve essential medical information without the introduction of image artifacts. Specifically, we investigated the GANs, CycleGAN, U-Net, cGAN, and our (LoHiResGAN) image-to-image translation model’s effectiveness in generating synthetic 3T MR images from the 64mT images. The brain morphometry was compared between the synthetic 3T images, the paired 3T, and the original 64mT images. Our findings indicate a high-level agreement between 3T and synthetic 3T brain morphometry measures, that provides more consistent results when compared with measures made using 3T and 64mT images.

## Materials and methods

### Data collection

Institutional ethics and institutional review board (IRB) approvals were obtained from Monash University Human Research Ethics Committee, and written informed consent was acquired from all participants involved in the study. All experiments were performed in accordance with relevant guidelines and regulations. The study, conducted between October 2022 and June 2023, involved 92 healthy individuals (mean age 44; range 18–81; SD = 17, 42 males). Each participant was scanned at Monash Biomedical Imaging using both Hyperfine Swoop (64mT) and Siemens Biograph mMR (3T) imaging systems. While the Hyperfine Swoop is designed to function in unshielded environments, leveraging its proprietary electromagnetic interference (EMI) removal technique, we encountered an EMI warning during our operations^5^. Consequently, we judiciously repositioned the scanner to a location where such warnings were no longer present. During recruitment, a 60-year-old female participant had a history of cerebral haemorrhage and subsequent development of a cerebrospinal fluid (CSF)-filled cavity, and this participant was excluded during model training but was subsequently used to test the model’s performance as an out-of-distribution sample. Table 1 provides the scanning parameters including voxel resolution, matrix size, and scan duration for both T1- and T2-weighted scans of both systems.

**Table 1:** Details of the scanning parameters for 64mT and 3T MRIs.

Scan type	Voxel resolution (mm$^3$)	Matrix size	Scan duration
64mT T1-weighted (AXI)	$1.60\times 1.60\times 5.00$	$112\times 138\times 36$	6.17 min
64mT T2-weighted (AXI)	$1.50\times 1.50\times 5.00$	$120\times 146\times 36$	6.30 min
3T T1-weighted MP-RAGE	$1.00\times 1.00\times 1.00$	$256\times 256\times 176$	5.30 min
3T T2-weighted TSE	$0.43\times 0.43\times 4.00$	$512\times 512\times 29$	1.50 min

In choosing the imaging sequences for this study, various factors were taken into consideration. For the Siemens Biograph mMR (3T) imaging system, we opted for a 2D T2-weighted TSE sequence over the more available 3D T2 SPACE. This decision was based on several grounds. First, the 2D T2-weighted TSE sequence offers faster scanning times compared to its 3D counterparts, ensuring efficiency and minimizing patient discomfort during imaging. Moreover, the 2D T2-weighted sequence is a cornerstone in many clinical protocols, and its results are well-established in the medical community^36^. Thus, leveraging this sequence ensured that our findings had immediate clinical relevance. Furthermore, the field of view for our 2D T2-weighted acquisition was meticulously crafted to align with the 64mT image acquisition, ensuring consistency across different imaging modalities within our study.

### Data pre-processing

The SynthSeg$^+$ method^34^ was used to resample the datasets to 1 mm$^3$ isotropic resolution, and FSL-FAST was used for bias field correction without referencing an external atlas for spatial information^16^. To prepare the data for deep learning methods, paired training was performed by co-registering the 64mT and 3T scans using FMRIB’s linear image registration tool (FLIRT)^37^. Finally, the dataset was randomly divided into training (n = 37), validation (n = 5), and testing (n = 50) sets, to ensure the network was trained, validated, and tested on different participant data. Also, where applicable, the relevant checklist for good machine learning practices (GMLPs) has been considered^38^. To prepare for the SynthSR^32^ method, we used the FLIRT registration method to co-register T1-w and T2-w images without resampling those to 1 mm$^3$ isotropic resolution. Once we had the SynthSR T1-w image, we resampled and co-registered with 3T (T1-w) images for further comparison.

### Model architecture

Our proposed method (LoHiResGAN, which signifies the low-field to high-field translation task and the use of ResNet components in a GAN architecture) was inspired by cGAN and Pix2Pix models, where the ResNet’s downsample and upsample blocks were used instead of standard U-Net^39^. The following Table 2 provides a detailed breakdown of the architectural components and their functions within the proposed LoHiResGAN model, highlighting the structure of both the generator and the discriminator. To empirically evaluate the effectiveness of the ResNet components in our LoHiResGAN model, we undertook experiments comparing its performance with architectures devoid of these components. Our findings supported the incorporation of ResNet, showing that its components contributed significantly to the improved translation of low-field MRI images to high-field MRI images. These experiments thereby validate the advantage of integrating ResNet components into our architecture.

**Table 2:** Summary of the Architecture of the LoHiResGAN Model: A ResNet-based Generative Adversarial Network (GAN) for Efficient Translation of Low-Field to High-Field MRI Images.

Component	Details
Generator	
Encoder (Downsampler)	
Layers	8
Filter sizes	64, 128, 256, 512, 512, 512, 512, 512
Components	Convolutional layers, batch normalization, ReLU activation, residual blocks
Function	Reduces spatial dimensions of the input image, increase the number of feature maps
Decoder (Upsampler)	
Layers	7
Filter sizes	512, 512, 512, 512, 256, 128, 64
Components	Transposed convolutional layers, batch normalization, ReLU activation, residual blocks, dropout layers (first three layers, dropout rate of 0.5)
Function	Increases spatial dimensions, decreases the number of feature maps
Output	Image of the same size as input, produced by a final transposed convolutional layer with a tanh activation function
Discriminator	
Architecture	PatchGAN-based
Function	Classifies whether input image patches are real or generated
Inputs	Input image, target image (same size)
Downsampling Layers	3
Filter sizes	64, 128, 256
Components	Convolutional layers, batch normalization (except the first layer), LeakyReLU activation functions
Function	Reduces spatial dimensions of input, increases the number of feature maps
Output	$30\times 30$ map, each value corresponds to classification of a $70\times 70$ patch in the input image

LoHiResGAN is a GAN-based architecture that leverages ResNet components for efficient translation of low-field MRI images to high-field MRI images, which achieves improved image quality and structural preservation. Replacement of the U-Net downsampling and upsampling blocks with the ResNet counterparts in a modified U-Net generator can improve performance by leveraging the ResNet ability to capture long-range dependencies and preserve fine-grained details.

As this study focused on translating low-field MRI to high-field MRI containing different domain information, we hypothesized that incorporation of the structural similarity index measure (SSIM) as an additional loss could provide more information for the overall loss calculation. While mean absolute error (MAE) and binary cross-entropy (BCE) losses measure different aspects of the generated output, they do not consider the perceptual similarity between the generated and target images. In contrast, the SSIM loss is a metric that captures the similarity between two images based on the luminance, contrast, and structure, and has been shown to be more closely aligned with human perception of image quality than traditional pixel-wise error metrics like MAE. By incorporation of SSIM as an additional loss term, the overall loss function considered not only the accuracy of the generated image but also its similarity to the target image in terms of structure and texture. This may lead to improved perceptual quality in the generated images. To test the efficacy of our approach, we conducted experiments comparing the results with and without the inclusion of SSIM loss. Our findings indicated that integrating SSIM into the loss function indeed favored the production of images with enhanced perceptual quality, validating our hypothesis and underscoring its potential utility in tasks involving image translation between domains.

1 $$\begin{aligned} \mathscr {L}_{LoHiResGAN}(G,D)= & {} \mathbb {E}_{x,y}[\log D(x,y)] + \mathbb {E}_{x,z}[\log (1-D(x,G(x,z))]. \end{aligned}$$

2 $$\begin{aligned} \mathscr {L}_{L1}(G)= & {} \mathbb {E}_{x,y,z}[||y - G(x,z)||_{1}]. \end{aligned}$$

3 $$\begin{aligned} \mathscr {L}_{SSIM}(G)= & {} \mathbb {E}_{x,y,z}[SSIM(y, G(x,z))]. \end{aligned}$$

4 $$\begin{aligned} \mathscr {T}= & {} \mathop {\min }\limits _G \mathop {\max }\limits _D {\mathscr {L}_{LoHiResGAN}}(G,D) + \lambda _1 \mathscr {L}_{L1}(G) + \lambda _2\mathscr {L}_{SSIM}(G). \end{aligned}$$

where *x* is the input image, *y* is the target image, *z* is the noise vector, *D* is the discriminator, *G* is the generator, $\lambda _1=100$ (as per the original Pix2Pix^40^) and $\lambda _2=1$ are used as the weighting factors for the $L_1$ loss and the SSIM loss respectively. $\mathscr {T}$ is the total objective function that aims to optimize during training.

In the context of the LoHiResGAN network, Eq. (1) represents the LoHiResGAN loss function that the generator, *G*, aims to minimize against an adversarial discriminator, *D*, which seeks to maximize it. This function comprises two components: one estimating the likelihood of the discriminator correctly identifying real image pairs and the other estimating the likelihood of identifying fake pairs generated by *G*. Equation (2) denotes the $L_1$ loss function, which measures the expected absolute difference between the target image, *y*, and the image produced by the generator, *G*(*x*, *z*). This function aims to bring the generated images closer to the target images. Equation (3) describes the Structural Similarity Index Measure (SSIM) loss for the generator, *G*. This metric calculates the expected structural similarity between the target and generated images, considering elements like structural information, luminance, and contrast. Finally, Eq. (4) encapsulates the overall objective function the LoHiResGAN seeks to optimize. This function is a combination of the LoHiResGAN loss, $L_1$ loss, and SSIM loss, weighted accordingly. The ultimate goal is to find the optimal *G* and *D* that minimize and maximizes these objectives, respectively.

We employed the end-to-end machine learning platform TensorFlow to perform training, validation, and testing. We applied several pre-processing steps to prepare the dataset for training different models, including input normalization within desired intensity ranges using minimum and maximum, random flipping, rotation and cropping. The Adam optimizer was selected based on its superior performance to other optimizers using the trial-and-error method. The training hyperparameters included a learning rate of $2e^{-3}$, $\beta _1=0.5$, $\beta _2=0.999$, 350 epochs, batch size 1, and shuffle after every epoch. Model training, testing and performance analysis were performed on a Ubuntu LTS (ver 20.04.5) operating system with the NVIDIA A40 GPU.

### Image quality assessment and statistical evaluation

The performance of image-to-image translation models was evaluated using quantitative metrics (Eqs. 5–7), namely, normalized root-mean-squared error (NRMSE), structural similarity index measure (SSIM), peak signal-to-noise ratio (PSNR), and perception-based image quality evaluator (PIQE)^41^. These metrics provide different perspectives on the quality of the predicted image relative to the 3T images. Post-processing steps (segmentation and statistical analysis) were employed to conduct a comparative analysis of brain morphometry across the 3T, 64mT, and synthetic 3T MRI scans. In addition to the previously mentioned metrics, the Sørensen-Dice similarity coefficient (DICE) was used to measure the overlap between the predicted segmentation and the ground truth (Eq. 8). This provides a quantitative evaluation of how accurate the image-to-image translation models are at preserving the morphological details of the structures of interest in the synthetic 3T MRI scans. The performance of these models was analyzed across the 3T, 64mT, and synthetic 3T MRI scans to conduct a comparative analysis of brain morphometry. The Dice coefficient can range from 0 to 1, where 1 indicates perfect overlap (i.e., the predicted segmentation is identical to the ground truth), and 0 indicates no overlap.

5 $$\begin{aligned} NRMSE(I, K)= & {} \frac{\sqrt{\frac{1}{N}\sum _{i=1}^{N}(I_i-K_i)^2}}{max(I) - min(I)} \end{aligned}$$

6 $$\begin{aligned} SSIM(I, K)= & {} \frac{(2\mu _I\mu _K + c_1)(2\sigma _{IK} + c_2)}{(\mu _I^2 + \mu _K^2 + c_1)(\sigma _I^2 + \sigma _K^2 + c_2)} \end{aligned}$$

7 $$\begin{aligned} PSNR(I, K)= & {} 20\log _{10}\left( \frac{MAX_I}{\sqrt{MSE(I,K)}}\right) \end{aligned}$$

8 $$\begin{aligned} DICE(A, B)= & {} \frac{2|A \cap B|}{|A|+|B|} \end{aligned}$$

where *I* and *K* are two vectorised, non-negative matrices representing two images of same size, and *N* is the number of elements in *I* or *K*. $\mu _I$ and $\mu _K$ are the average of *I* and *K*, respectively; $\sigma _{I}$ and $\sigma _{K}$ are the standard deviation of *I* and *K*; $\sigma _{IK}$ is the covariance of *I* and *K*. $c_1=(k_1L)^2$ and $c_2=(k_2L)^2$ are two variables to stabilize the division with weak denominator; *L* is the dynamic range of the pixel-values. For PIQE, consider the original article^41^. *A* and *B* are, respectively, the set of pixels in the predicted segmentation and the ground truth. $|A \cap B|$ is the cardinality of the intersection of *A* and *B*. |*A*| and |*B*| are the cardinalities of *A* and *B*, respectively.

In the present investigation, skull-stripping was conducted using a brain extraction tool as reported by Jenkinson et al.^42^. Specifically, T1-weighted images from 3T were employed to generate binary brain masks for subsequent scans. Also, the one-way ANOVA tests conducted to compare the brain volume measurements of 33 regions between the original 3T, 64mT, SynthSR, and LoHiResGAN scans. The one-way ANOVA test was chosen due to its suitability for multiple group comparisons, mild assumptions (normality tested by Shapiro-Wilk and equal variance tested by Levene’s tests), simultaneous group comparison capability, and provision for effect size and post-hoc analysis. Finally, the findings were statistically analysed by using the Tukey HSD (honestly significant difference) test, which was performed to conduct post-hoc pairwise comparisons between the means (33 regions) of different MRI modalities (3T, 64mT, SynthSR, and LoHiResGAN) and control the family-wise error rate.

## Results

### Image quality metrics

Table 3 compares the overall performance of various image-to-image translation models on two different MRI sequences, T1- and T2-weighted. This comparison is based on four key metrics: NRMSE, PSNR, SSIM, and PIQE. LoHiResGAN displays the lowest NRMSE and PIQE values and the highest PSNR and SSIM values, outperforming other methods across all metrics.

**Table 3:** Comparison of normalized root-mean-squared error (NRMSE), peak signal-to-noise ratio (PSNR), structural similarity index measure (SSIM), and perception-based image quality evaluator (PIQE) with other state-of-the-art methods for T1- and T2-weighted (mean ± standard deviation).

Sequence	Method	NRMSE$\downarrow$	PSNR$\uparrow$	SSIM$\uparrow$	PIQE$\downarrow$
T1	64mT	$0.432\pm 0.025$	$21.638\pm 1.148$ dB	$0.856\pm 0.015$	$71.820\pm 1.969$
	GANs	$0.140\pm 0.029$	$31.330\pm 1.381$ dB	$0.948\pm 0.004$	$60.896\pm 1.791$
	CycleGAN	$0.132\pm 0.022$	$31.820\pm 1.262$ dB	$0.948\pm 0.003$	$62.130\pm 1.851$
	U-Net	$0.126\pm 0.009$	$30.683\pm 1.210$ dB	$0.936\pm 0.005$	$61.423\pm 1.729$
	cGAN	$0.125\pm 0.023$	$32.302\pm 1.529$ dB	$0.946\pm 0.004$	$62.479\pm 1.731$
	LoHiResGAN	${\textbf {0.104}}\pm {\textbf {0.007}}$	${\textbf {33.842}}\pm {\textbf {1.311}}$ dB	${\textbf {0.959}}\pm {\textbf {0.004}}$	${\textbf {55.293}}\pm {\textbf {1.476}}$
T2	64mT	$0.379\pm 0.026$	$22.289\pm 0.406$ dB	$0.883\pm 0.012$	$69.366\pm 1.999$
	GANs	$0.115\pm 0.012$	$32.319\pm 1.019$ dB	$0.954\pm 0.003$	$54.532\pm 1.261$
	CycleGAN	$0.112\pm 0.011$	$32.457\pm 0.885$ dB	$0.948\pm 0.003$	$56.773\pm 1.242$
	U-Net	$0.131\pm 0.010$	$31.558\pm 0.542$ dB	$0.941\pm 0.004$	$57.185\pm 1.539$
	cGAN	$0.113\pm 0.011$	$32.977\pm 0.967$ dB	$0.954\pm 0.003$	$56.570\pm 1.058$
	LoHiResGAN	${\textbf {0.110}}\pm {\textbf {0.010}}$	${\textbf {33.127}}\pm {\textbf {0.824}}$ dB	${\textbf {0.965}}\pm {\textbf {0.003}}$	${\textbf {55.336}}\pm {\textbf {1.193}}$

### Qualitative image comparison

**Figure 1:**
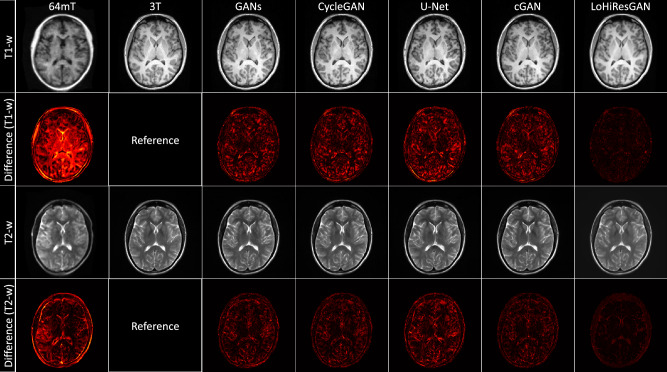
Comparison of T1-weighted and T2-weighted images (32-year-old male participant) and their absolute difference (plotted in the range of [0, 1]) with respect to 3T reference images across various state-of-the-art image-to-image translation techniques. From left to right: 64mT, 3T, GANs, CycleGAN, U-Net, cGAN, and LoHiResGAN.

Figure 1 shows substantial qualitative disparities between representative 64mT and 3T images in both T1-weighted and T2-weighted modalities. These visual differences among the 64mT, GANs, CycleGAN, U-Net, and cGAN images are minimized by LoHiResGAN. An absolute difference image is also computed for each method’s output, demonstrating the variation in translation results between these methods. This shows the residual signal error compared to 3T and is clearly lowest for LoHiResGAN.

**Figure 2:**
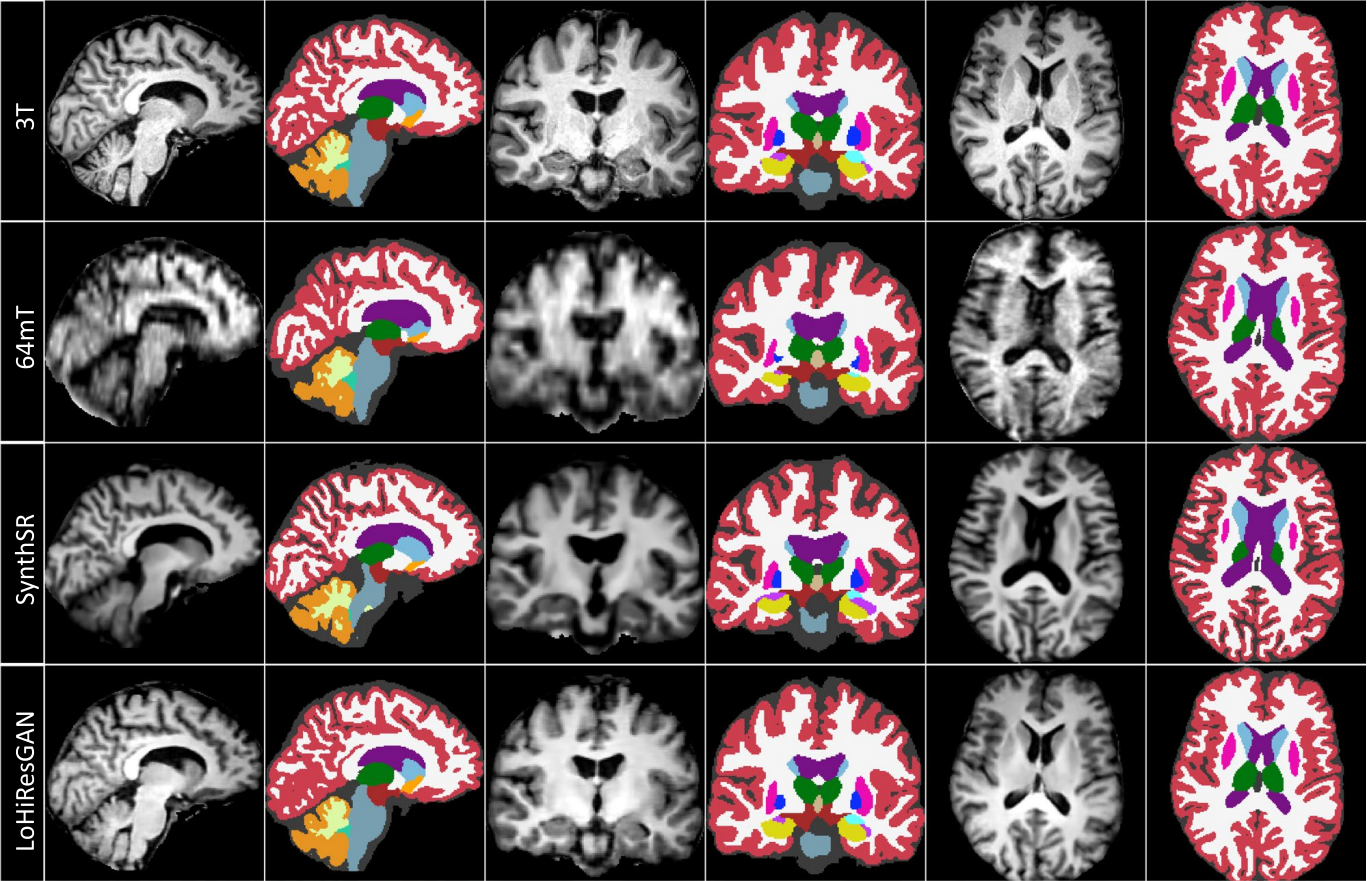
Brain regions visualised using orthogonal views (T1-weighted): Sagittal, Coronal, and Axial for 3T (first row), 64mT (second row), SynthSR (third row), and LoHiResGAN (fourth row).

Brain parcellation (by SynthSeg$^+$) for different methods in 33 brain regions is shown in Fig. 2. The SynthSeg$^+$ model is publicly accessible and designed for user-friendly interaction. We used the pre-trained SynthSeg$^+$ model to evaluate our proposed method’s performance without any additional fine-tuning on our low-field MRI data. Qualitative analysis of segmented masks for 64mT, 3T, SynthSR, and LoHiResGAN brain regions shows large errors in CSF segmentation of the lateral ventricles, Sylvian fissures, and sulci at 64mT. Differences are also evident in the hippocampus, cerebral white matter, cortex, and deep brain structures with erroneous labeling between grey and white matter. Whilst SynthSR improves the visual appearance, there is significant smoothing and no improvement in the segmentation of CSF spaces. The segmentation of LoHiResGAN shows high visual similarity to that of 3T.

### Quantitative regional brain volume

**Figure 3:**
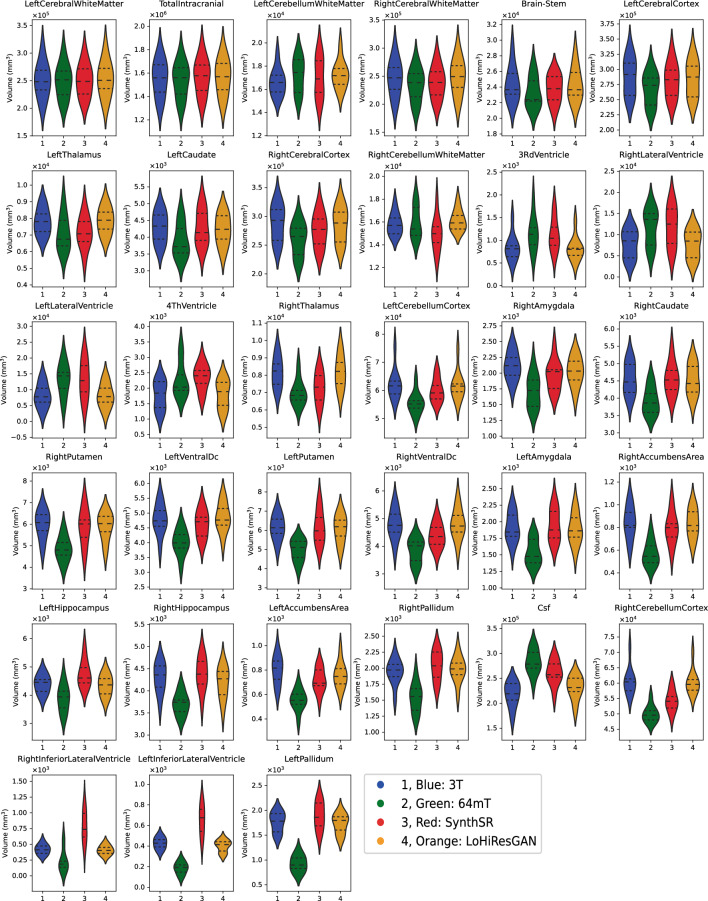
Comparative analysis of brain volume measurements (33 regions) across 3T (1, blue), 64mT (2, green), SynthSR (3, red) and LoHiResGAN (4, orange).

Figure 3 compares brain volume measurements for four different datasets: 3T, 64mT, SynthSR, and LoHiResGAN. Each plot corresponds to one of the 33 brain regions analyzed, with the width of each plot indicating the data distribution and the mean and inner quartiles marked by horizontal lines. In reference to 3T images, the 64mT images demonstrate large errors across multiple brain regions with differences in mean volume for cerebral cortex ($\approx$ 30% underestimate), hippocampus ($\approx$ 30% underestimate), and CSF ($\approx$ 30% overestimate), whereas changes of only $\approx$5.6-6.8% have been shown to have clinical significance in patients with hydrocephalus. SynthSR images show marked improvements in quantitative brain volume accuracy in all brain regions compared to the 64mT images. In comparison, LoHiResGAN consistently improves the accuracy across all brain regions. Specifically, LoHiResGAN reduces the underestimation observed in regions like the ventricle, cerebellum cortex, and hippocampus, while also attenuating the overestimation present in white matter (WM) and CSF.

The Tukey HSD test of the difference in mean volumes is presented in Table 4. There is a statistically significant difference in mean volumes when comparing 3T versus 64mT and also comparing 3T versus SynthSR measurements. However, there is no statistically significant difference in volumes comparing 3T versus LoHiResGAN measurements, confirming their close similarity. The test provides mean differences, adjusted *p*-values, and confidence intervals and indicates whether the null hypothesis is rejected for each pairwise comparison.

**Table 4:** Tukey HSD test results.

Group 1	Group 2	Mean Diff.	*p*-adj.	Lower bound	Upper bound	Reject
3T	64mT	− 981.5509	0.0002	− 1537.5353	− 425.5665	True
3T	LoHiResGAN	− 382.2771	0.2688	− 938.2615	173.7073	False
3T	SynthSR	16.4986	0.9998	− 539.4857	572.483	False
64mT	LoHiResGAN	599.2738	0.0303	43.2895	1155.2582	True
64mT	SynthSR	998.0495	0.0001	442.0652	1554.0339	True
LoHiResGAN	SynthSR	398.7757	0.2351	− 157.2086	954.7601	False

**Figure 4:**
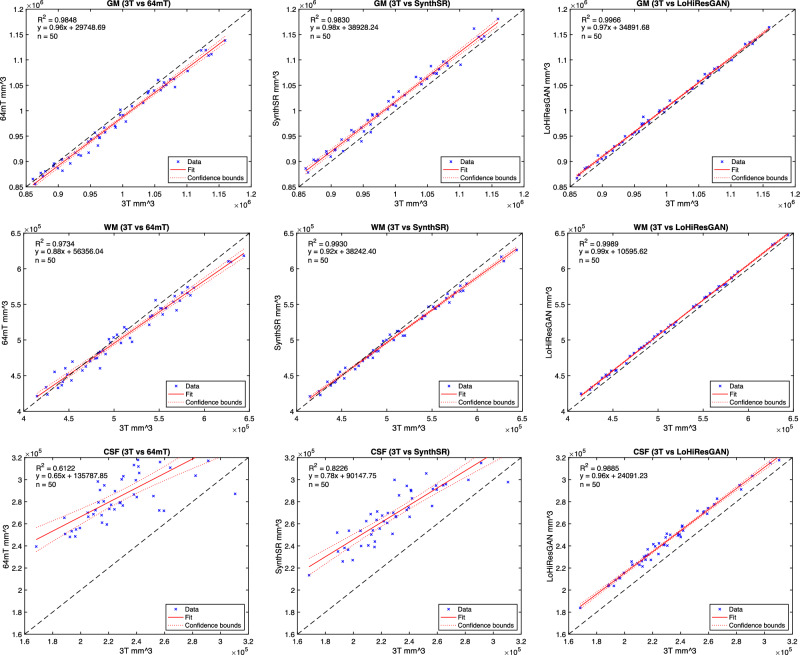
Comparative linear regression analysis of GM, WM, and CSF measurements among 3T, 64mT, SynthSR, and LoHiResGAN MRIs with 50 observations. Slope equations are as follows: GM measurements: 3T vs. 64mT ($y = 0.96x + 29748.69$), 3T vs. SynthSR ($y = 0.98x + 38928.24$), 3T vs. LoHiResGAN ($y = 0.97x + 34891.68$). WM measurements: 3T vs. 64mT ($y = 0.88x + 56356.04$), 3T vs. SynthSR ($y = 0.92x + 38242.40$), 3T vs. LoHiResGAN ($y = 0.99x + 10595.62$). CSF measurements: 3T vs. 64mT ($y = 0.65x + 135787.85$), 3T vs. SynthSR ($y = 0.78x + 90147.75$), 3T vs. LoHiResGAN ($y = 0.96x + 24091.23$). The $R^2$ values and the slope equations emphasize the precision and reliability of the LoHiResGAN method.

Further, we employ a linear regression model to ascertain the measured volumetric accuracy from different imaging techniques, specifically focusing on gray matter (GM), WM, and CSF (Fig. 4). The comparison is made among four methodologies: 3T, 64mT, SynthSR, and LoHiResGAN. When comparing the CSF measurements, the relationship between 3T and 64mT yields an $R^2$ value of 0.6122, and for SynthSR, an $R^2$ value of 0.8226, marking a notable moderate to strong linear correspondence. The comparative analysis between 3T and LoHiResGAN results in an $R^2$ value of 0.9885, emphasizing an excellent linear association. Delving deeper into GM measurements, the interrelation between 3T and 64mT shows a significant $R^2$ value of 0.9848, and for SynthSR, the $R^2$ value is 0.9830. This association is further strengthened in the 3T and LoHiResGAN proximity, which registers an $R^2$ value of 0.9966. Turning to WM measurements, a robust linear correlation is observed between 3T and 64mT with an $R^2$ of 0.9734 and an $R^2$ of 0.9930 for SynthSR. Notably, this relationship reaches its peak when 3T is paired with LoHiResGAN, yielding an $R^2$ of 0.9989. These findings collectively underscore the precision and reliability inherent in the LoHiResGAN method.

### Quantitative segmentation

**Figure 5:**
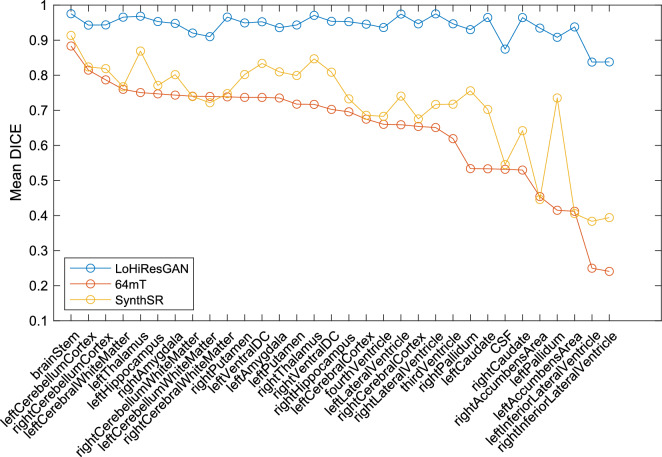
Comparison of mean DICE scores for synthetic 3T MRI (LoHiResGAN), 64mT MRI, and SynthSR segmentations across different brain regions versus 3T ground truth segmentation (without total ICV). Note, higher DICE scores indicate better agreement with the 3T ground truth segmentation.

For structural overlap of the segmentations, the DICE similarity coefficient between the reference (3T) segmentation and the segmentations generated using 64mT, SynthSR, and LoHiResGAN images is shown in Fig. 5 for each brain region across all subjects. Using LoHiResGAN, the DICE similarity coefficient for synthetic 3T improves compared to the original 64mT across all brain regions, achieving scores mostly $>0.9$ (where 1 indicates perfect agreement) and shows notably substantial improvement in important clinical and research regions such as the cerebral cortex, hippocampus, and CSF. SynthSR alone shows milder improvement in DICE similarity coefficient and critically performs worse than native 64mT for CSF volume. The observed variability in DICE scores across different brain regions can be attributed to several factors, including the inherent complexity of the region’s anatomy, tissue contrast with surrounding tissues, and the effectiveness of the segmentation algorithm in delineating the boundaries of these structures. The Dice coefficient is highly related to a structure’s size given its sensitivity to errors at the surface of a structure^43^. As such, smaller structures tend to show lower Dice coefficients, limiting its use when comparing between different structures. The quantitative analysis of the mean DICE scores highlights the improved segmentation quality achieved by the synthetic 3T MRI compared to the 64mT MRI.

**Figure 6:**
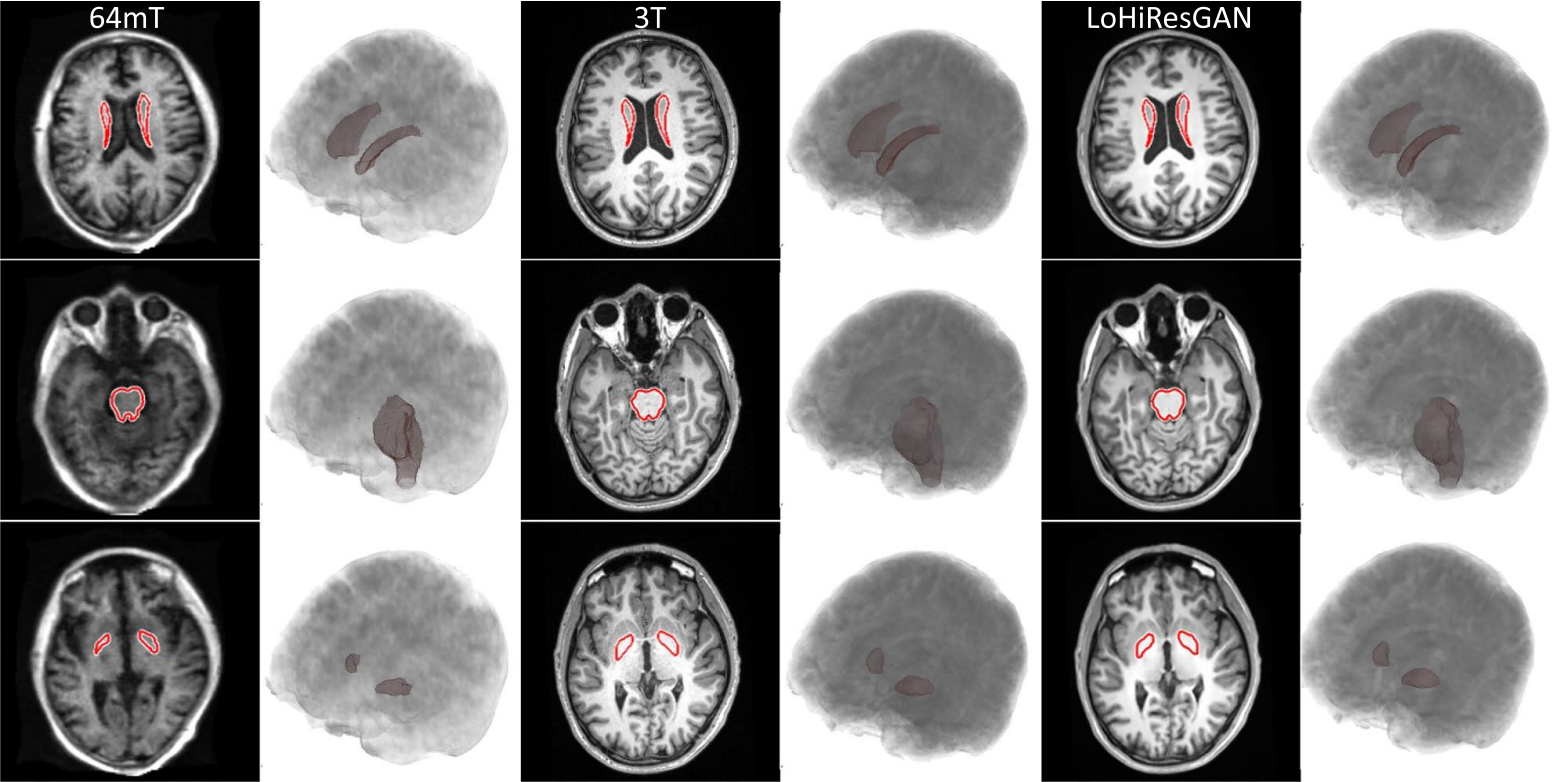
T1-weighted images showcasing the caudate nucleus, brain stem, and globus pallidus in rows one, two, and three, respectively, with their 3D renderings from a 26-year-old male.

In Fig. 6, T1-weighted images provide a detailed visualization of three specific neural regions-the caudate nucleus (depicted in the first row), brain stem (shown in the second row), and globus pallidus (featured in the third row). Their 3D renderings, based on data from a 26-year-old male participant, further elucidate their structural nuances. A pronounced disparity is evident in the pallidum region between the 64mT and LoHiResGAN images. In contrast, the brain stem and caudate showcase minimal differences across the modalities. Importantly, when focusing on image quality, LoHiResGAN images bear a closer resemblance to the original 3T images, underlining their diagnostic potential.

**Figure 7:**
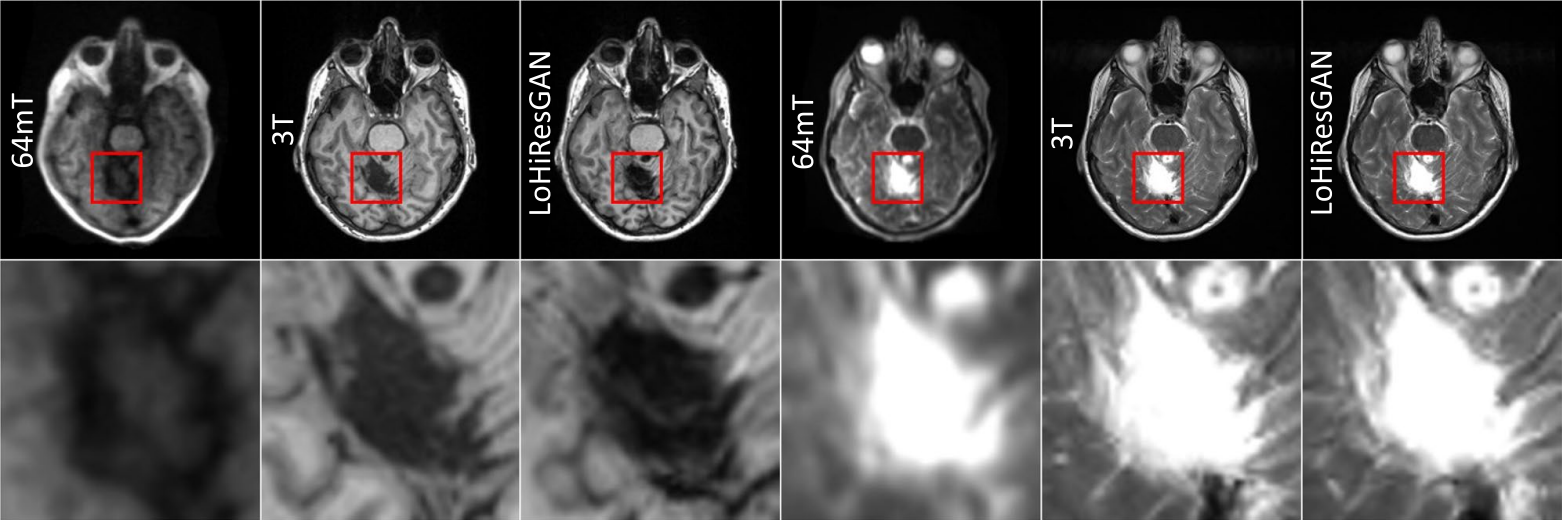
T1-weighted images (columns 1-3) and T2 weighted images (columns 4-6) using 64mT MRI scan, 3T MRI scan, and LoHiResGAN respectively in a 60-year-old female with focal encephalomacia. The second row of images displays the region of interest extracted from corresponding images.

In addition to the in-distribution testing, we further explore the performance of the models in out-of-distribution scenarios, specifically focusing on the ability to capture and represent abnormal pathological areas. Preliminary results reveal that the abnormal pathological areas are clearly discernible in both T1-weighted and T2-weighted sequences of the synthetic 3T images. This is showcased in Fig. 7, which demonstrates superior consistency between the synthetic images and the corresponding acquired 3T images.

## Discussion

This study presents a new deep learning-based image-to-image translation model, LoHiResGAN, to enhance the quality of low-field (64mT) MRI scans and generate synthetic high-field (3T) MRI scans from low-field scans.

Comparative assessment of multiple state-of-the-art deep learning-based methods for T1-weighted and T2-weighted image quality assessments shows all achieve significantly better SNR and SSIM than the original 64T scans. GAN-based methods, including LoHiResGAN, outperform the UNet model in including NRMSE, PSNR, SSIM, and PIQE metrics, particularly for T2-weighted images. This superior performance highlights the potential of deep learning techniques, particularly GAN-based methods like LoHiResGAN, for improving image quality of low-field MRI.

Brain regions show discrepancies in volume measurements at 64mT when compared to the reference 3T scans. Such discrepancies highlight the challenges faced in relying solely on low-field MRI scans for clinical diagnosis and planning. For instance, the overestimation of certain measurements in 64mT images may have significant clinical implications, especially in conditions like hydrocephalus. The need for more accurate reconstruction models is evident, emphasizing the potential role of deep learning solutions in improving the image quality of low-field MRI scans. LoHiResGAN, as one such solution, appears promising in addressing some of these challenges. However, it’s crucial to acknowledge that no method is infallible. Despite the improvements brought about by LoHiResGAN, there remains a spectrum of inconsistencies it has yet to fully address. This suggests that there is room for further optimization and refinement. Interestingly, while some imaging methods seem to align in their representation of particular brain regions, detailed examination brings to light potential mislabeling issues, such as the misidentification of grey matter and white matter. Such nuances underscore the challenges in achieving a truly accurate representation of brain anatomy through imaging. It’s imperative for future research to focus on these challenges. By refining these methods, we can ensure that they not only improve the visual quality of MRI scans but also provide data that clinicians and researchers can rely upon for accurate interpretations and decisions.

The results highlight the efficacy of LoHiResGAN in enhancing the DICE similarity coefficient for synthetic 3T MRI, particularly when compared against the original 64mT MRI. This is especially pertinent in regions pivotal for both clinical diagnoses and research, like the cerebral cortex, hippocampus, and CSF. While SynthSR’s capabilities seem to be more moderate, it’s concerning that it sometimes underperforms compared to the native 64mT, raising questions about its utility for certain applications. The variability in DICE scores across regions underscores the multifaceted challenges in MRI segmentation. The anatomy’s inherent complexity, the varied tissue contrasts, and the proficiency of the segmentation algorithms all play roles in these discrepancies. Notably, the Dice coefficient’s particular sensitivity to structural boundaries, especially for smaller anatomical features, reminds the need for caution when employing this metric across diverse structures. Further, evaluations on varying orientations observed consistent results. Such insights emphasize the importance of refining segmentation tools and methodologies, particularly as we navigate the nuances of different brain structures. The benefits of synthetic 3T MRI show that tools like LoHiResGAN could be beneficial when traditional methods are not available, emphasizing the importance of further development in this area.

The out-of-distribution test in a patient with focal encephalomalacia highlights the potential of advanced image-to-image translation techniques to enhance diagnostic accuracy in pathologic cases, despite the model being exclusively trained on data from healthy subjects. Notably, however, the signal of the abnormality in this case remains CSF. Further research involving a more extensive and diverse dataset, including patients with various neurological pathologies, is needed to determine the clinical utility, accuracy, and ultimately contribute to the development of more robust image-to-image translation models.

Despite the encouraging findings, our proposed study has limitations. Generalisability is constrained by the relatively small sample size, which consists solely of healthy subjects. This is especially true for patterns not within the training data set. Nonetheless, for the purposes of volumetry, the sample spans patients with a broad range of ages and parenchymal volumes. A smoothing effect emerges during the analysis of generated images and may diminish the ability to resolve small structures. The underlying performance of the image segmentation method itself is not taken into account when conducting volumetric comparisons in our study. This oversight can lead to biased segmentation outcomes, although the automated results are concordant with visual inspection. Future research should address these limitations to further enhance the validity and reliability of the study findings.

Several future directions are worth exploring to enhance further the performance of image-to-image translation models for low-field to high-field MRI translation. Firstly, incorporating larger and more diverse datasets, including individuals with various neurological disease conditions, will help improve the generalizability and robustness of the model for clinical diagnostic purposes. This would ultimately contribute to better diagnostic accuracy and more targeted treatment planning in clinical settings. Secondly, exploring the combination of multiple image-to-image translation models, or even developing novel models tailored to specific brain regions or conditions, may yield further improvements in image quality and accuracy of brain morphometry measurements for different experimental settings. Lastly, integrating advanced generative deep learning techniques such as transformer models, diffusion deep learning models, and unsupervised learning approaches could potentially enhance the performance of image-to-image translation models in low-field to high-field MRI translation tasks by capturing more complex patterns and dependencies in the data.

In conclusion, the findings of this study demonstrate the substantial clinically significant limitations in native 64mT data and suggest that the application of image-to-image translation models, such as LoHiResGAN, can substantially improve the quality with synthetic high-field images approaching 3T quality. If shown to be reproducible across a range of brain pathologies, this will have significant implications for clinical and research settings, particularly in resource-limited settings where access to high-field MRI scanners may be limited. The spectrum of findings emphasizes the importance of considering similarity across different brain regions when evaluating the performance of image translation models. Further investigation into the factors contributing to regional discrepancies will enhance our understanding of the challenges associated with accurate brain structure measurements. Finally, by further exploring and refining these models, we can continue advancing the medical imaging field and contribute towards a more accurate and reliable assessment of brain structures and functions.

## Data Availability

The datasets generated and/or analysed during the current study are not publicly available due to confidentiality agreements with participants but are available from the corresponding author upon reasonable request. The code used for our tests is publicly available at https://github.com/khtohidulislam/LoHiResGAN for evaluation.
